# *RFC1*-related ataxia is a mimic of early multiple system atrophy

**DOI:** 10.1136/jnnp-2020-325092

**Published:** 2021-02-09

**Authors:** Roisin Sullivan, Wai Yan Yau, Viorica Chelban, Salvatore Rossi, Natalia Dominik, Emer O'Connor, John Hardy, Nicholas Wood, Andrea Cortese, Henry Houlden

**Affiliations:** 1 Neuromuscular Diseases, UCL Queen Square Institute of Neurology, London, UK; 2 The National Hospital for Neurology and Neurosurgery, London, UK; 3 Institute of Neurology, Policlinico Universitario Agostino Gemelli, Roma, Italy; 4 Molecular Neuroscience, UCL, Queen Square, Intitute of Neurology, London, UK; 5 Clinical and Movement Neurosciences, UCL Queen Square Institute of Neurology, London, UK

**Keywords:** multisystem atrophy, neurogenetics, cerebellar ataxia

## Introduction

Multiple systems atrophy (MSA) is a rare neurodegenerative disorder combining varying presentations of cerebellar impairment, parkinsonism and autonomic dysfunction that relies on pathological examination for a definitive diagnosis.^1 2^ The second consensus diagnostic criteria (SCDC) subdivide patients into two groups based on a predominance of either parkinsonism (MSA-P) or cerebellar impairment (MSA-C).^2^ MSA is frequently misdiagnosed, especially early in the disease course, with only 60% of possible and probable MSA meeting the pathological criteria.^2 3^

A recessive intronic expansion in replication factor C subunit 1 (*RFC1*) has been found to be a cause of late-onset ataxia and cerebellar ataxia, neuropathy and vestibular areflexia syndrome (CANVAS),^4^ Due to the overlapping clinical features of MSA and CANVAS, we hypothesised that the recessive *RFC1* expansion may be implicated in patients that atypically fit the diagnostic criteria for MSA. Here, we present three cases with biallelic *RFC1* expansions: two clinically diagnosed with ‘probable’ MSA and one with ‘probable’ based on the SCDC for MSA.^2^

## Methods

### Samples

We enrolled 207 patients with ‘possible’ and ‘probable’ MSA, from the National Hospital for Neurology and Neurosurgery (NHNN). MSA diagnosis based on the SCDC for MSA.^2^

### Genetic testing

**Figure 1:**
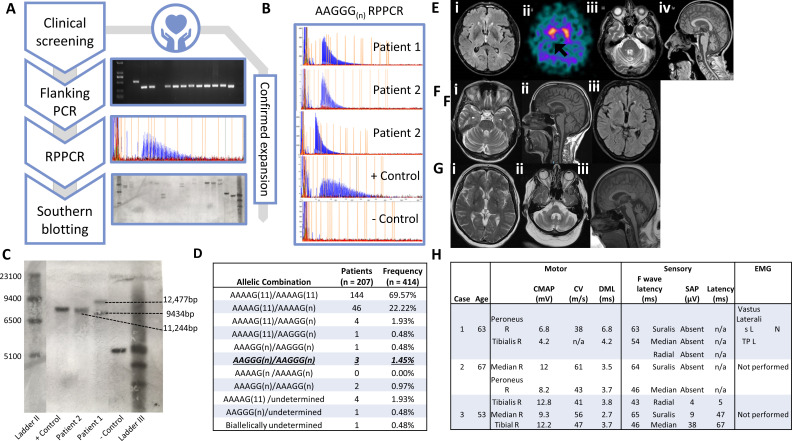
Clinical presentation of affected patients. (A) Workflow of *RFC1* screen; clinical screening of cohort leads to sample gDNA undergoing flanking PCR and those with no amplifiable product and potential carriers are taken forward for RPPCR. Samples with a positive RPPCR trace (hallmark sawtooth pattern) are then Southern blotted, confirming size of expansion. (B) CANVAS RPPCR positive fragment analysis traces for patients 1, 2 and 3 and a positive CANVAS and negative control. (C) Southern blot results with size of bands indicated; 12 477 and 9434 bp for patient 1 which encompass 953 and 1541 repeat units, 11 244 bp for patient 2 (1315 repeat units) (black dashed lines), 11 244 bp for positive CANVAS control and 5000 bp for negative control. (D) Allelic carrier frequency of different allelic combinations in clinical MSA cohort. Known pathogenic conformation *AAGGG/AAGGG* underlined. (E) Neuroimaging of patient 1 at age 66 years. (i) MRI head T2 FLAIR axial sequence showed minimal changes in basal ganglia, (ii) DaTscan showed asymmetrical reduction in putaminal dopamine transporter uptake (black arrow), (iii) MRI head T2 axial sequence showed pontine and middle cerebellar peduncle atrophy without ‘hot cross bun’ sign and (iv) MRI head T1 sagittal sequence shows cerebellar and medulla atrophy. (F) Neuroimaging of patient 2 at age 65 years. (i) MRI head T2 FLAIR axial sequence showed normal pontine appearance, (ii) MRI head T1 sagittal sequence showed no evidence of cerebellar or brainstem atrophy and (iii) MRI head T2 FLAIR axial sequence showed normal appearance of basal ganglia. (G) Neuroimaging of patient 3 at age 56 years. (i) MRI head T2 axial sequence showed symmetrical diminution of lentiform nuclei with subtle hyperintensity along dorsolateral aspect of the putamen, (ii) MRI T2 axial sequence showing ‘hot cross bun’ sign and (iii) MRI T1 axial sequence showed pontine and cerebellar atrophy. (H) NCS showed sensory neuronopathy with normal motor studies and mild distal denervation on EMG while for patient 1 and patient 2 showed sensory neuronopathy with normal motor studies. Patient 3 was normal. (11), wild type conformation; bp, base pairs; CANVAS, cerebellar ataxia, neuropathy and vestibular areflexia syndrome; CMAP, compound motor action potential; DML, distal motor latency; EMG, electromyography; gDNA, genomic DNA; MCV, motor conduction velocity; MSA, multiple systems atrophy; MUP, motor unit potentials; N, normal; (n), expanded conformation; NCS, nerve conduction study; RBD, REM sleep behaviour disorder; REM, rapid eye movement; RPPCR, repeat primed PCR; SAP, sensory action potential; TP, tibialis posterior.

Genomic DNA was extracted from blood and investigated under approval of the joint ethics committee of the UCL Institute of Neurology and The NHNN, London, UK (UCLH: 04/N034). Flanking PCR, repeat-primed PCR and Southern blot were performed on all samples as previously described^4^ (figure 1A).

## Results

Three patients had biallelic *RFC1* expansions, as defined by no amplifiable product on PCR, and a positive repeat primed PCR for AAGGG_(n)_ (figure 1A, B). The allelic frequencies in clinical MSA cases were 1.7% (n=7) for pathogenic AAGGG_(n)_, 2.4% (n=10) for AAAGG_(n)_, 11.4% (n=47) for AAAAG_(n)_ and 82.8% (n=343) for AAAAG_(11)_ (figure 1D). Southern blotting revealed two large expansions, sized at 9434 kb (887 repeat units) and 12 477 kb (1495 repeat units) in patient 1 (figure 1C) and 11 244 kb (1249 repeat units) in patient 2, corresponding to the two expanded alleles; both patients were initially diagnosed with ‘possible’ MSA. There was insufficient DNA to perform a Southern blot for patient 3, initially diagnosed with ‘probable’ MSA.

### Case studies

Patient 1 has possible MSA: symptom onset at 62 years old, autonomic tests within 3 years of disease onset showed mild cardiovascular autonomic dysfunction, levodopa non-responsive parkinsonism and a cerebellar syndrome (online supplemental video 1). He progressed to wheelchair use at 67 years, complicated by neurogenic bladder and presyncope. He had no preceding chronic cough or pins and needles. His brother also had ataxia, attributed to alcohol abuse and died at 62 years old. Examination at 69 years old revealed a combination of parkinsonism and cerebellar ataxia with orthostatic blood pressure drop >30/15 mm Hg. His strength was retained but reflexes were brisk with absent ankle jerks and absent plantar response. Pinprick was absent throughout with asymmetrical impairment of vibration to trunk and reduced proprioception distally in the lower limbs. Neuro-otological review showed no vestibular areflexia. Routine investigations for ataxia and genetic tests (SCA 1, 2, 3, 6, 7, 12, 17, FRDA and whole exome sequencing) were negative. Figure 1E and H show his MRI head, DaTscan and nerve conduction study (NSC).

10.1136/jnnp-2020-325092.supp1 Supplementary video

Patient 2 has possible MSA: symptom onset at 62 years old, orthostatic decrease of blood pressures was <30 mm/15 mm Hg with symptoms of unexplained bladder urgency, levodopa non-responsive parkinsonism and a cerebellar syndrome (online supplemental video 2). Her initial symptoms were postural dizziness, gait imbalance and cough with rapid decline developing dysphagia, dysarthria and mild neurogenic bladder over 2 years. Family history was not contributory. On examination at age 66, she had a stooped posture and narrow-based gait with loss of bilateral arm swing. VORs were impaired on bedside testing. In additional to cerebellar ataxia, finger tapping was slow and showed decrements with interruptions. Her overall strength was preserved with absent plantar reflex. Her other reflexes were symmetrical and brisk. Sensory examination revealed loss of vibration to ankles and absent pinprick sensation in lower limbs and distal upper limbs. Routine investigations for ataxia were normal. Genetic tests were negative. Figure 1F and H showed the results of her MRI head and NSC.

10.1136/jnnp-2020-325092.supp2 Supplementary video

Patient 3 has probable MSA: symptom onset at age 53 years old, orthostatic decrease of blood pressures was >30 mm/15 mm Hg, levodopa non-responsive parkinsonism and a cerebellar syndrome. Her initial clinical manifestations were gait imbalance, dysarthria and increased extrapyramidal tone in her right hand. Her family history was not contributary. On examination at age 56, she had a broad-based gait, head drop and axial flexion deformity. She was bradykinetic with increased tone and reduced tempo in her right hand but no increased tone in her lower limbs. with a slight reduction in her left. She also had finger dysmetria bilaterally with impaired tandem gait. Her reflexes were reduced in the lower limbs with upgoing plantar responses bilaterally. Autonomic testing showed parasympathetic impairment, with postural blood pressure drop, unexplained urinary urgency and incomplete bladder emptying. NCS results were normal (figure 1H). MRI head showed extensive atrophy of the superior and middle cerebellar peduncles, cerebellum and pons (figure 1G). Genetic tests were negative.

## Discussion

We have identified three patients that have biallelic *RFC1* repeat expansions, presenting with a combination of cerebellar ataxia and parkinsonism: two initially diagnosed with possible MSA and one with probable MSA. This expands the spectrum of *RFC1* related disease that can range from idiopathic late onset ataxia through to CANVAS, but is absent in neuropathologically confirmed MSA cases.^5^ We suggest that the phenotype includes mimics of MSA-C and likely other atypical parkinsonian phenotypes. If these cases come to autopsy, we would not expect to see MSA pathology.

These patients present with an MSA mimic in the early stage, but slower clinical and radiological progression suggest that they are not phenotypically representative of MSA. Sensory neuropathy is always difficult to assess in MSA, given the frequent cold and swollen feet with dependent oedema.

Given the difficulty in diagnosing MSA in the early stages, we recommend testing for the *RFC1* expansion in atypical MSA and parkinsonian patients, particularly in those with atypical features such as peripheral neuropathy, slow disease progression, impaired vestibulo-ocular reflex and clinically mild autonomic dysfunction.
